# Clinical Outcome and Prognostic Factors of Intensity-Modulated Radiotherapy for T4 Stage Nasopharyngeal Carcinoma

**DOI:** 10.1155/2016/4398498

**Published:** 2016-04-19

**Authors:** Yangkun Luo, Yang Gao, Guangquan Yang, Jinyi Lang

**Affiliations:** ^1^Department of Radiation Oncology, Sichuan Cancer Hospital, Chengdu 610041, China; ^2^Department of Radiation Oncology, Zigong No. 4 People's Hospital of Sichuan Province, Zigong 643000, China

## Abstract

*Objective*. To analyze the clinical outcomes and prognostic factors of intensity-modulated radiotherapy (IMRT) for T4 stage nasopharyngeal carcinoma (NPC).* Methods*. Between March 2005 and March 2010, 110 patients with T4 stage NPC without distant metastases were treated. All patients received IMRT. Induction and/or concurrent chemotherapy were given. 47 (42.7%) patients received IMRT replanning.* Results*. The 5-year local recurrence-free survival (LRFS), regional recurrence-free survival (RRFS), distant metastasis-free survival (DMFS), progression-free survival (PFS), and overall survival (OS) rates were 90.1%, 97.0%, 67.5%, 63.9%, and 64.5%, respectively. Eleven patients experienced local-regional failure and total distant metastasis occurred in 34 patients. 45 patients died and 26 patients died of distant metastasis alone. The 5-year LRFS rates were 97.7% and 83.8% for the patients that received and did not receive IMRT replanning, respectively (*P* = 0.023). Metastasis to the retropharyngeal lymph nodes (RLN) was associated with inferior 5-year OS rate (61.0% versus 91.7%, *P* = 0.034). The gross tumor volume of the right/left lymph nodes (GTVln) was an independent prognostic factor for DMFS (*P* = 0.006) and PFS (*P* = 0.018). GTVln was with marginal significance as the prognostic factor for OS (*P* = 0.050).* Conclusion*. IMRT provides excellent local-regional control for T4 stage NPC. Benefit of IMRT replanning may be associated with improvement in local control. Incorporating GTVln into the N staging system may provide better prognostic information.

## 1. Introduction

Nasopharyngeal carcinoma (NPC) is a common malignancy in southern China and radiotherapy (RT) is the primary treatment [1]. The five-year overall survival (OS) rate for early-stage NPC has exceeded 90% following treatment with RT alone [2]. However, it is important to note that the majority of patients present with locoregionally advanced stages of NPC at the time of diagnosis. According to the American Joint Committee on Cancer (AJCC) 2010 staging system [3], T4 stage NPC is characterized as a tumor with intracranial extensions and/or involvement of the cranial nerves, hypopharynx, orbit, and extensions to the infratemporal fossa/masticator space. RT of T4 stage NPC represents a great challenge due to dose limitation requirements for critical structures such as the spinal cord and brainstem.

Intensity-modulated radiotherapy (IMRT) has replaced two-dimensional RT due to its dosimetric advantages. The use of IMRT and chemotherapy in combination has also been found to provide excellent locoregional control [4–6]. Unfortunately, however, improvement in locoregional control has not been accompanied by an increase in long-term OS. While studies of concurrent chemoradiation [7–11] which have included different combinations of chemotherapy and radiation have shown high rates of locoregional control, OS and particularly distant metastasis-free survival (DMFS) rates remain suboptimal. Furthermore, data regarding the clinical outcome of T4 stage NPC cases are relatively rare.

Adaptive radiotherapy (ART) has also been studied in the setting of IMRT for head and neck cancers. For this treatment approach, RT plan is modified in response to anatomic and dosimetric changes during treatment in order to ensure adequate target coverage and to improve quality of life by reducing the treatment dose to normal tissue [12–16]. To date, many studies have focused on dosimetric analyses, and limited data have been published regarding the effect of ART on clinical outcome. In the present study, treatment results from cases of T4 stage NPC were retrospectively analyzed and both clinical outcome and prognostic factors were examined. In addition, the potential clinical benefit of adaptive IMRT replanning is discussed.

## 2. Materials and Methods

### 2.1. Pretreatment Evaluations of the Patients Selected

Between March 2005 and March 2010, 110 patients that were newly diagnosed with T4 stage NPC and were treated at the Sichuan Cancer Hospital were enrolled in this study. The pretreatment workup performed for each patient included a complete history and physical examination, complete blood counts, blood chemistries, an endoscopy, magnetic resonance imaging (MRI) of the nasopharynx and neck, chest computed tomography (CT) or radiography, an abdominal ultrasound, and emission computed tomography (ECT). The inclusion criteria were as follows: (1) pathologically confirmed NPC, (2) receiving radical IMRT treatment, (3) a Karnofsky performance score > 70, (4) receiving chemotherapy, (5) complete RT plan documentation, (6) an absence of pregnancy or lactation, and (7) an absence of previous malignancy or other concomitant malignant disease. Patients with distant metastasis at diagnosis and those who did not complete a full course of RT were excluded. All patients underwent disease restaging according to the AJCC 2010 staging system [3] and patient characteristics for the present cohort are summarized in Table 1. This study was approved by the ethics committee of Sichuan Cancer Hospital.

### 2.2. RT

All patients were treated with IMRT with 6-megavoltage (MV) photons. Target volumes were in agreement with the International Commission on Radiation Units and Measurements Reports 50 and 62 [17, 18]. RT planning was designed and optimized using the CORVUS 3.4–4.2 inverse treatment planning system (Peacock, Nomos, Deer Park, IL, USA). The gross tumor volume of the nasopharynx (GTVnx) and gross tumor volume of the right/left lymph nodes (GTVln) were outlined based on CT and MRI scans. Clinical target volume 1 (CTV1) included the GTVnx with a 5–10 mm margin and high risk structures. Clinical target volume 2 (CTV2) potentially involved regions including the nasopharyngeal cavity, the maxillary sinus, the pterygopalatine fossa, the posterior ethmoid sinus, the parapharyngeal space, the skull base, the anterior third of the clivus, the inferior sphenoid sinus, and the cavernous sinus. The clinical target volume of the right/left lymph nodes (CTVln) included the lymphatic drainage regions (the bilateral retropharyngeal nodes and levels II, III, and V_A_). The prescribed radiation doses were defined as follows: 66–76 Gy for GTVnx, 60–70 Gy for GTVln, 60–66 Gy for CTV1, 54–60 Gy for CTV2, and 50–54 Gy for CTVln using a simultaneous integrated boost technique, each divided into 30–33 deliveries. In addition, the dose limits for each normal organ were set according to the Radiation Therapy Oncology Group protocol 0225 [5]. The prescribed dose encompassed at least 95% of the target volume, and no greater than 1% of the nasopharynx gross target volume received ≤93% of the prescribed dose. The maximum dose of the treatment plan was applied within the target volume.

The IMRT plan was applied via dynamic intensity-modulated coplanar arc irradiation using a MIMI multileaf collimator (NOMOS Corporation, Sewickley, PA, USA). For all of the patients, radiation was applied to the lymph node drainage areas in the lower neck by using ^60^Co split field techniques or 6 MV X-ray split-beam techniques, with a prescription dose of 50 Gy in 25 fractions. Positive cervical lymph nodes in the lower neck received a total dose of 60–70 Gy by electron beam boost irradiation.

For this study, target volumes (including GTVnx and GTVln) were measured by the treatment planning system. The system automatically calculated the volume by the summation of area technique. Because the split-beam technique was used in this study, the low neck positive cervical lymph nodes were not included in the initial IMRT plan. These positive cervical lymph nodes were contoured according to the description of the boost plan. Then the whole volumes of positive cervical lymph nodes were recalculated by the system.

### 2.3. IMRT Replanning

A total of 47 (42.7%) patients received 1–4 IMRT replans (median: 3). The decision to replan was made at the physician's discretion and multiple factors were considered: weight loss, nutritional status, changes in palpable or visible tumor size, an ill-fitting mask, and the extent of acute radiation reactions. When a tumor was close to the spinal cord or brainstem and other important organs, replanning was typically needed early in the intervention process, and additional replans could be made if needed. If a dose escalation was needed, a replan was routinely administered and a new CT scan would be performed. During each CT scan, the patient maintained the same position and the new CT scan was used to generate a new IMRT plan for the corresponding fractions of treatment. To ensure relative consistency of target delineation, a CT-CT fusion was used by rigid registration and was adjusted manually according to the region of interest. GTVnx and GTVln, along with the organs at risk, were contoured on the new CT scans. The CTV was maintained and modified according to the changes in anatomic structure that occurred. The time from resimulation to implementation of the new IMRT plan was generally 1–3 days. The first IMRT replan was implemented at a median dose of 44 Gy (range, 8.8–57.2).

### 2.4. Chemotherapy

All patients received platinum-based chemotherapy and did not receive adjuvant chemotherapy. The induction chemotherapy regimens included TP (75 mg/m^2^ docetaxel and 80 mg/m^2^ cisplatin on day 1) or PF (100 mg/m^2^ cisplatin on day 1 and 1000 mg/m^2^/day 5-fluorouracil for days 1–5) every three weeks for 1-2 cycles. Concurrent chemotherapy included 80 mg/m^2^ cisplatin every three weeks for 2-3 cycles. A total of 27 (24.5%) patients received targeted therapy including cetuximab or nimotuzumab.

### 2.5. Patient Assessments and Follow-Up

All patients were evaluated weekly during RT and then they underwent follow-up upon completion of RT, as well as 1 month after the completion of RT, every 3 months in the first 2 years, every 6 months from year 3 to year 5, and annually thereafter. Each follow-up included a complete examination involving a flexible fiberoptic endoscopy, an ultrasound of the abdomen, a chest X-ray, and basic serum chemistry. Either CT or MRI of the head and neck was performed after the completion of IMRT and then was performed every 6 months from then on. Treatment-related toxicities were assessed according to the National Cancer Institute Common Toxicity Criteria (NCI-CTC) version 3.0 [19]. RT-related toxicities were graded according to RTOG criteria [20].

### 2.6. Statistical Analysis

All analyses were performed with SPSS, version 16.0 (IBM, Chicago, IL, USA). Local recurrence-free survival (LRFS), regional recurrence-free survival (RRFS), DMFS, progression-free survival (PFS), and OS were estimated according to the Kaplan-Meier method. LRFS, RRFS, DMFS, PFS, and OS were measured from day 1 of treatment to the date of the event. For the univariate and multivariate analyses performed, the log-rank test and the Cox proportional hazards model were employed, respectively. A *P* value less than 0.05 was considered to be statistically significant.

## 3. Results

### 3.1. Treatment Outcome

**Figure 1:**
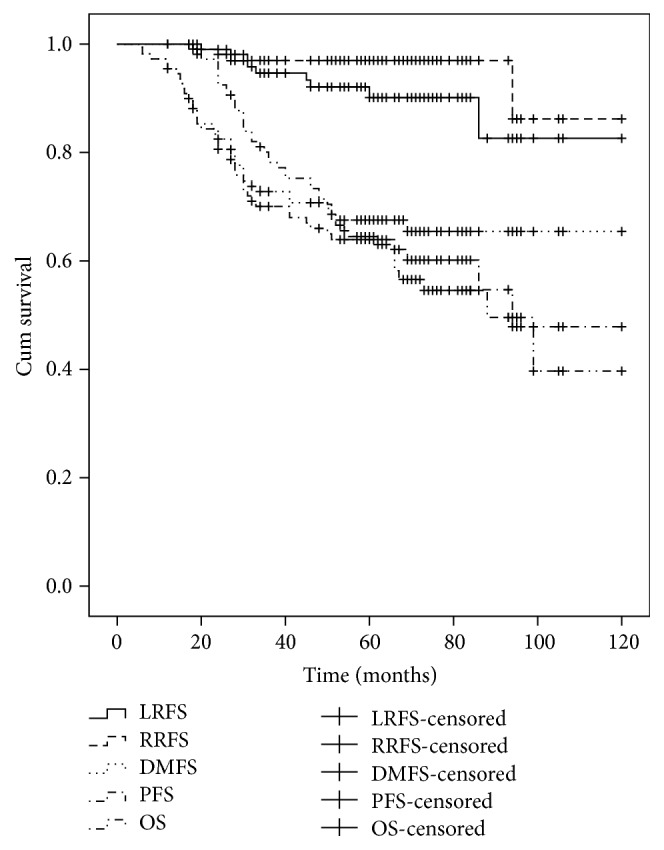
Kaplan-Meier curves for LRFS, RRFS, DMFS, PFS, and OS for the present cohort.

The median follow-up time for the present cohort was 58 months (range, 12–120 months). The overall 5-year LRFS, RRFS, DMFS, PFS, and OS rates were 90.1%, 97.0%, 67.5%, 63.9%, and 64.5%, respectively (Figure 1).

**Figure 2:**
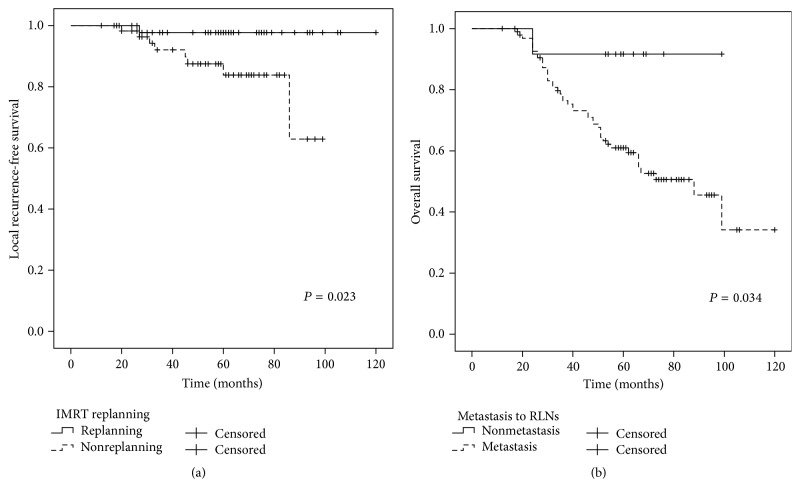
(a) Comparison of the 5-year LRFS curves for the patients that were treated with and without IMRT replanning. Log-rank test; *P* = 0.023. (b) Comparison of the 5-year OS curves for the patients with or without metastasis to the RLN. Log-rank test; *P* = 0.034.

The 5-year LRFS rates for patients with or without IMRT replanning were 97.7% and 83.8%, respectively (*P* = 0.023) (Figure 2(a)). In addition, the 5-year DMFS, PFS, and OS rates for the patients with or without IMRT replanning were 71.1% and 65.9%, 69.4% and 56.7%, and 67.2% and 62.6%, respectively, in each case (Table 2).

**Figure 3:**
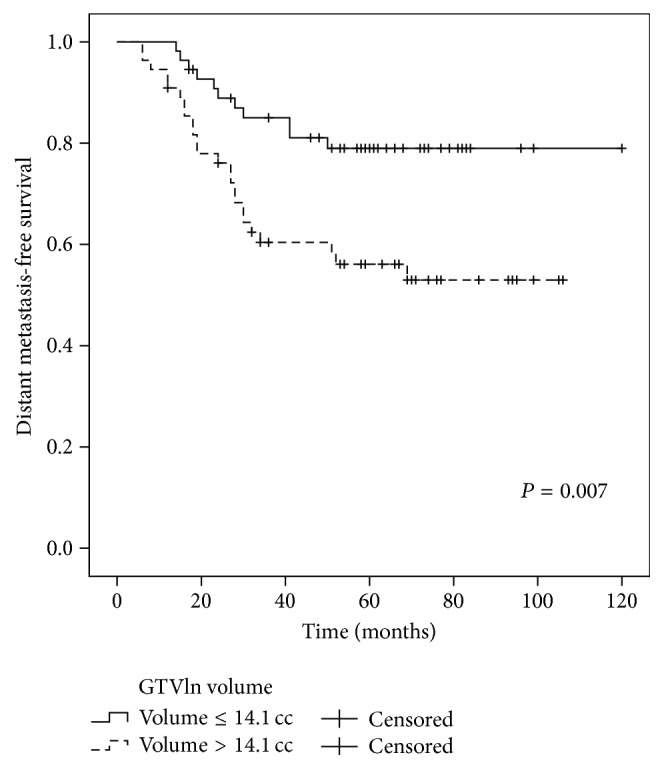
Comparison of the 5-year DMFS curves for the patients with a GTVln ≤ 14.1 cc versus a GTVln > 14.1 cc. Log-rank test; *P* = 0.007.

**Figure 4:**
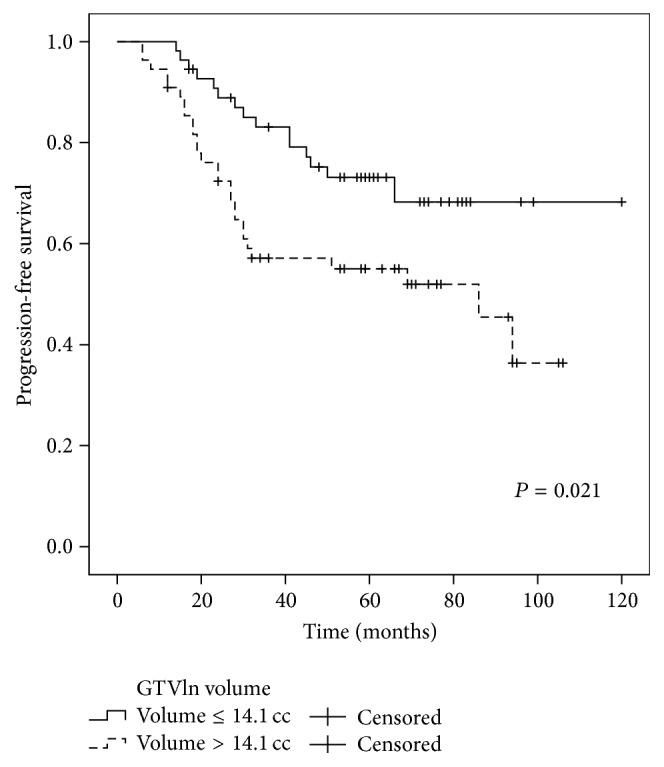
Comparison of the 5-year PFS curves for the patients with a GTVln ≤ 14.1 cc versus a GTVln > 14.1 cc. Log-rank test; *P* = 0.021.

**Figure 5:**
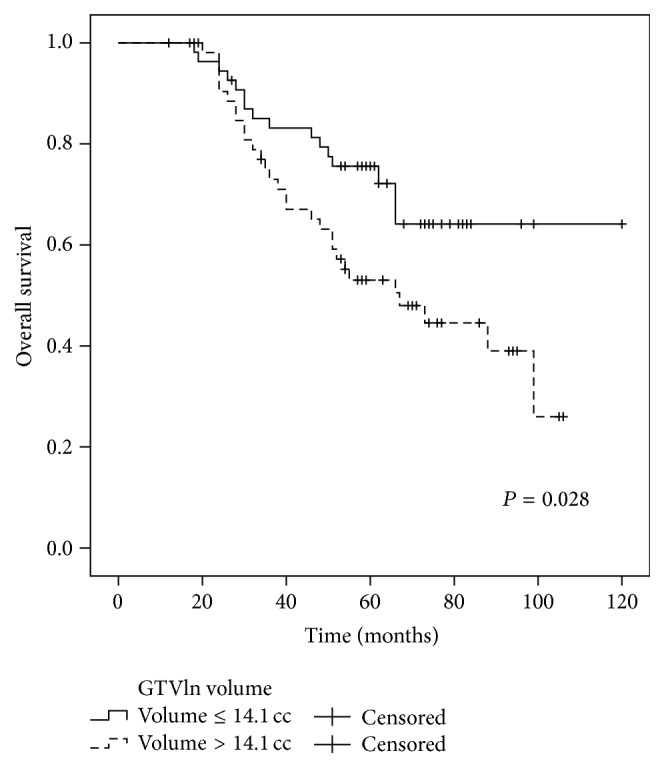
Comparison of the 5-year OS curves for the patients with a GTVln ≤ 14.1 cc versus a GTVln > 14.1 cc. Log-rank test; *P* = 0.028.

The 5-year OS rates for the patients with or without metastasis to the retropharyngeal lymph nodes (RLN) were 61.0% and 91.7%, respectively (*P* = 0.034) (Figure 2(b)). The 5-year DMFS, PFS, and OS for patients with GTVln ≤ 14.1 cc and GTVln > 14.1 cc were 79.0% and 56.1% (*P* = 0.007), 73.1% and 55.0% (*P* = 0.021), and 75.6% and 53.0% (*P* = 0.028), respectively (Figures 3–5). There were no statistically significant differences in the LRFS, RRFS, DMFS, and OS rates in the GTVnx, cervical nodal necrosis, targeted therapy, and induction or concurrent chemotherapy. A total of 45 patients died due to severe bleeding of the nasopharynx (*n* = 5), severe bleeding of the nasopharynx combined with locoregional recurrence (*n* = 1), unknown internal disease (*n* = 5), locoregional recurrence (*n* = 5), locoregional recurrence with distant metastasis (*n* = 3), and distant metastasis (*n* = 26). Furthermore, a total of 41 failures were observed that involved distant metastasis alone (*n* = 30), local recurrence alone (*n* = 5), cervical lymph node relapse alone (*n* = 2), local recurrence and distant metastasis (*n* = 3), and cervical lymph node relapse and distant metastasis (*n* = 1).

### 3.2. Prognostic Factors

The value of various potential prognostic factors, including age, gender, N stage, GTVnx, GTVln, chemotherapy, IMRT replanning, targeted therapy, metastasis to the RLN, and cervical nodal necrosis, for predicting LRFS, RRFS, DMFS, PFS, and OS was evaluated. Univariate analysis showed that GTVln was significantly associated with DMFS, PFS, and OS, while IMRT replanning was significantly associated with LRFS.

There was no difference in DMFS, PFS, and OS for the patients with or without IMRT replanning (*P* = 0.743, *P* = 0.302, and *P* = 0.646, resp.) (Table 2).

Furthermore, metastasis to the RLN was significantly associated with OS (Table 2).

In multivariate analysis, GTVln, IMRT replanning or not, and metastasis to the RLN or not which were proved by univariate analysis to have the potential affecting survival rate were included in the Cox proportional hazard model. The results showed that GTVln was an independent negative prognostic factor of DMFS and PFS (HR = 2.718, 95% CI: 1.324–5.577, and *P* = 0.006 and HR = 2.159, 95% CI: 1.141–4.088, and *P* = 0.018, resp.). The GTVln also exhibited marginal significance as a prognostic factor for OS (HR = 1.875, 95% CI: 1.001–3.512, and *P* = 0.050).

### 3.3. Toxicity

All patients tolerated the entire treatment regimen. The main manifestations of radiation-related acute toxicity included xerostomia, mucositis, and dermatitis. The acute toxicities were controllable and were mainly characterized as grades 1-2. However, grade 3 xerostomia, mucositis, and dermatitis were observed, with rates of 4.5% (*n* = 5), 22.7% (*n* = 25), and 6.4% (*n* = 7), respectively. None of the patients experienced grade 4 toxicity. Regarding hematologic toxicity, 10 (9.1%) patients experienced grade 4 leukopenia, while liver and kidney dysfunction were mainly grades 1-2. Late onset toxicities were assessed for all of the 110 patients at least one year after the completion of treatment. The incidence of grades 0-1, 2, 3, and 4 xerostomia was 73.6% (*n* = 81), 23.6% (*n* = 26), 2.7% (*n* = 3), and 0.0%, respectively. Only one patient that underwent IMRT replanning developed grade 3 xerostomia, and there were no cases of radiation-induced cranial nerve palsy. There were 13 patients that developed radiation-induced temporal lobe necrosis that was diagnosed by MRI during the follow-up period. Four of these patients (4/47; 8.5%) underwent IMRT replanning and nine of these patients (9/63; 14.3%) did not undergo IMRT replanning.

## 4. Discussion

IMRT has replaced two-dimensional RT for the treatment of NPC because it delivers a higher radiation dose to a tumor while sparing the adjacent organs at risk. This can potentially enhance treatment outcome, especially the local control rate. In a study by Chen et al. [21], the 5-year OS, local relapse-free, and DMFS rates for 140 patients with T4 stage NPC that were treated by IMRT were 69.3%, 84.9%, and 73.6%, respectively. In another study [22] of 335 T4 stage NPC patients, the 5-year local failure-free survival (LFFS), regional failure-free survival (RFFS), distant failure-free survival (DFFS), and OS rates were 84.1%, 92.2%, 74.1%, and 63.0%, respectively. Sun et al. [6] reported that 5-year disease-specific survival (DSS), DMFS, LRFS, and PFS rates were 70.7%, 71.7%, 83.3%, and 59.8%, respectively. Similar 5-year rates for LRFS, RRFS, DMFS, PFS, and OS were observed in the present study (e.g., 90.1%, 97.0%, 67.5%, 63.9%, and 64.5%, resp.).

Distant metastasis was the main pattern of failure and the major cause of death in the present study. A total of 11 patients experienced local-regional failure and total distant metastasis occurred in 32 patients. There were 26 patients that died of distant metastasis alone. These results are similar to those reported by other studies of IMRT [5, 6, 23–25]. Therefore, although IMRT has been reported to provide excellent local-regional control, an improvement in OS was not observed as well. It is well known that N stage is related to distant metastasis in NPC patients. Previously, N status was found to be a significant predictor for distant failure and survival [26]. N status usually considers the size of a node, the node level, extranodal neoplastic spread (ENS), nodal necrosis, metastasis to the RLN, and other factors [6, 26–29]. In the univariate analysis performed in the present study, metastasis to the RLN was significantly associated with OS (*P* = 0.034). Cervical nodal necrosis also exhibited a marginal value for DMFS and PFS (*P* = 0.065 and *P* = 0.064, resp.) and N stage was a marginal predictor for OS (*P* = 0.087). In the multivariate analysis, GTVln was an independent negative prognostic factor of DMFS and PFS (*P* = 0.006 and *P* = 0.018, resp.), while GTVln exhibited marginal significance as a prognostic factor for OS (*P* = 0.050). Based on these results, it would appear that tumor burden is more important than N stage alone. Thus, while IMRT can achieve excellent local control, it does not eliminate micrometastatic lesions that are present prior to treatment, and this is consistent with the high local control and low DMFS and OS rates that characterize this treatment regimen. Furthermore, based on the present results, incorporating GTVln into the N staging system for T4 stage NPC may increase the prognostic value of this parameter, and this should be further studied.

Severe bleeding of the nasopharynx is a fatal complication after radiotherapy. The reason may be related to nasopharynx necrosis. When the necrotic lesion invades carotid artery, especially internal carotid artery, leading to rupture of the artery wall, it can cause severe bleeding [30]. T stage, radiation dose, and course of irradiation were the reasons of the nasopharynx necrosis [31]. In the present study, 5 patients died due to severe bleeding of the nasopharynx. For these patients, high total radiation dose (>70 Gy) and fraction dose (>2.2 Gy) were given. Moreover, 2 patients with IMRT replanning were treated with higher radiation dose (≥74 Gy). In addition, the parapharyngeal space was invaded and the internal carotid artery was enclosed by the primary tumor. These may lead to higher dose on the soft tissue and internal carotid artery, increased risk of necrosis, and severe bleeding of the nasopharynx. So, more caution should be considered in the dose distribution for these patients.

Compared with conventional two-dimensional RT, IMRT has not improved distant control for N stage tumors. Thus, the survival benefits of IMRT for NPC patients may solely derive from improved local control [32]. In recent years, ART has been evaluated as an IMRT treatment. The purpose of ART is to ensure adequate target coverage and to improve quality of life by reducing the radiation dose that is applied to normal tissues [12–16]. To correct for variations in tumors and normal tissues, ART is used to modify RT plan at the corresponding time points based on the acquisition of online or offline images. In the present study, adaptive IMRT replanning was considered based on an offline image that was obtained at our hospital. The first IMRT replan was implemented with a median dose of 44 Gy (range, 8.8–57.2). There were a total of 47 (42.7%) patients that received 1–4 IMRT replans (median: 3). While there was no standard for determining whether the replanning and the intervention time were successful, the results obtained show that IMRT replanning achieved better local control compared with non-IMRT replanning (97.7% versus 83.8%, resp.; *P* = 0.023), yet the rates of DMFS, PFS, and OS were not statistically significant. The latter results are also consistent with those of previous studies [12, 13, 16].

The treatment-related toxicities observed in the present study were well tolerated by the patients involved, and this has been observed in previous studies [6, 22]. In particular, it was observed that the patients who underwent IMRT replanning had a lower incidence of temporal lobe necrosis compared with the patients that did not undergo IMRT replanning (8.5% versus 14.3%, resp.). However, a direct comparison is not entirely appropriate due to the imbalance in the distribution of patients that did or did not undergo IMRT replanning, although this result may partly explain the theory of the association of improved quality of life associated with IMRT replanning that was proposed by Yang et al. [13]. To our knowledge, the present study is one of the first studies to suggest that the benefits of adaptive IMRT replanning may be associated with an actual clinical advantage, including improved local control and late toxicities such as temporal lobe necrosis for T4 stage NPC patients. However, it is important to note that the present study is limited by a small sample size. Therefore, the benefit of IMRT replanning remains to be confirmed with a larger sample size. Furthermore, prospective randomized trials are needed to identify specific predictive factors and to determine the criteria for adaptive IMRT replanning for NPC patients.

Currently, the treatment for locoregional advanced NPC includes concurrent chemotherapy/RT with or without adjuvant chemotherapy and induction chemotherapy followed by chemotherapy/RT. It has been hypothesized that induction chemotherapy is able to target a greater number of local and distant metastasis tumor cells, while also decreasing radiation side effects by reducing tumor burden. In the present study, there was no statistically significant difference between induction chemotherapy and concurrent chemotherapy in relation to LRFS, RRFS, DMFS, PFS, and OS. There was also no statistically significant difference in the use of targeted therapy or not. However, in a recent meta-analysis [33], induction chemotherapy was found to significantly reduce the hazard of progression and distant metastasis in cases of local advanced NPC on the basis of concurrent chemoradiotherapy. Another treatment regimen for local advanced NPC involves concurrent chemo/RT followed adjuvant chemotherapy. In a Bayesian network meta-analysis [34], however, adjuvant chemotherapy did not appear to improve patient survival following chemoradiotherapy. Meanwhile, a MAC-NPC meta-analysis [35] demonstrated that the benefit of a concomitant plus adjuvant chemotherapy schedule produced the greatest tumor-related outcomes compared with other treatment modalities. Thus, the results for induction or adjuvant chemotherapy for the treatment of NPC vary and are contradictory. Therefore, it remains for the treatment modalities for T4 stage NPC to be further studied.

There are several limitations associated with the present study, including the retrospective nature of the study design and the small number of studied patients. Possible prognostic factors, such as involvement of cranial nerves and the extent of intracranial involvement, also were not studied because of insufficient medical records or image data. These limitations may have potentially affected the outcomes observed. In addition, dosimetry analysis and evaluation of quality of life were not included in the present study. However, all of the patients in the present cohort received platinum-based chemotherapy and popular treatment modalities, and a potential benefit of IMRT replanning associated with an actual clinical advantage of local control for T4 stage NPC was observed.

In conclusion, the results of the present study demonstrate that IMRT can provide excellent local-regional control for T4 stage NPC, and IMRT replanning may further improve local control. The incorporation of GTVln into the N staging system may also provide better prognostic information. With distant metastasis observed to be the major cause of treatment failure in the present study, treatment modalities that effectively reduce the rate of distant metastasis and increase the survival rate of T4 stage NPC patients still need to be explored.

## Figures and Tables

**Table 1:** Characteristics of 110 patients with T4 stage NPC.

Patient characteristics	Number of patients (%)
Age (y)	
Range	17–69
Median	44.5
Gender	
Male	87 (79.1)
Female	23 (20.9)
N stage	
N0-1	35 (31.8)
N2-3	75 (68.2)
GTVnx (cc)	
Range	22.37–246.10
Median	77.27
GTVln (cc)	
Range	0–180.15
Median	14.1
IMRT replanning	
No	63 (57.3)
Yes	47 (42.7)
Chemotherapy	
Concurrent	75 (68.2)
Induction plus concurrent	35 (31.8)
Targeted therapy	
No	83 (75.5)
Yes	27 (24.5)
Metastasis to RLN	
No	13 (11.8)
Yes	97 (88.2)
Cervical nodal necrosis	
No	68 (61.8)
Yes	42 (38.2)

GTVnx, gross tumor volume of the nasopharynx; GTVln, gross tumor volume of the lymph nodes; IMRT, intensity-modulated radiotherapy; RLN, retropharyngeal lymph nodes.

**Table 2:** Impact of prognostic factors on treatment according to univariate analysis (log-rank test).

Characteristics	LRFS	*P*	RRFS	*P*	DMFS	*P*	PFS	*P*	OS	*P*
Age (yrs)										
≤44.5 yrs	89.4	0.743	96.0	0.895	62.4	0.418	63.9	0.876	63.5	0.813
>44.5 yrs	90.4		98.0		73.1		63.8		65.4	
Gender										
Male	90.0	0.334	96.2	0.331	62.4	0.418	63.9	0.876	63.0	0.493
Female	91.1		100.0		73.1		63.8		69.6	
N stage										
N0-1	91.9	0.807	100.0	0.110	75.8	0.339	75.9	0.272	79.4	0.087
N2-3	89.4		95.6		64.1		58.7		57.3	
GTVnx (cc)										
≤77.27	85.7	0.161	97.8	0.728	68.4	0.386	61.1	0.274	66.6	0.584
>77.27	95.4		96.1		70.3		67.0		62.4	
GTVln (cc)										
≤14.1	89.5	0.693	98.1	0.382	79.0	0.007	73.1	0.021	75.6	0.028
>14.1	91.3		95.7		56.1		55.0		53.0	
Chemotherapy										
Concurrent	90.8	0.788	97.0	0.431	71.6	0.214	68.6	0.149	70.2	0.250
Induction plus concurrent	89.3		97.0		58.8		54.1		52.6	
IMRT replanning										
No	83.8	0.023	96.2	0.853	65.9	0.743	56.7	0.302	62.6	0.646
Yes	97.7		97.9		71.1		69.4		67.2	
Targeted therapy										
No	87.8	0.308	97.6	0.339	69.6	0.532	64.7	0.941	62.6	0.841
Yes	96.2		100.0		61.5		61.5		70.0	
Metastasis to RLN										
No	90.9	0.941	100.0	0.468	91.7	0.075	83.3	0.122	91.7	0.034
Yes	90.1		96.6		64.3		61.4		61.0	
Cervical nodal necrosis										
No	92.1	0.181	97.0	0.660	73.7	0.065	71.0	0.064	71.6	0.112
Yes	86.3		96.7		57.3		52.0		51.6	
